# Development of the Emirates Multi-Criteria Decision Analysis Tool for Orphan Drugs

**DOI:** 10.7759/cureus.55215

**Published:** 2024-02-29

**Authors:** Khalid A Alnaqbi, Baher Elezbawy, Ahmad N Fasseeh, Abdul Rauf Bangash, Amin Elshamy, Hiba Shendi, Mohammed I Aftab, Mozah AlMarshoodi, Nicole Gebran, Noura AlDhaheri, Sahar A Fahmy, Sara Al Dallal, Waeil Al Naeem, Sherif Abaza, Zoltán Kaló

**Affiliations:** 1 Internal Medicine, College of Medicine & Health Sciences, United Arab Emirates University, Al Ain, ARE; 2 Internal Medicine/Rheumatology, Tawam Hospital, Al Ain, ARE; 3 Evidence Synthesis, Syreon Middle East, Alexandria, EGY; 4 Doctoral School of Pharmaceutical Sciences, Semmelweis University, Budapest, HUN; 5 Modelling, Syreon Middle East, Alexandria, EGY; 6 Faculty of Pharmacy, Alexandria University, Alexandria, EGY; 7 Benefit Design and Strategic Purchasing, Department of Health, Abu Dhabi, ARE; 8 Wellbeing and Sustainable Development, Ministry of Health and Prevention, Dubai, ARE; 9 Pediatrics, Tawam Hospital, Al Ain, ARE; 10 Procurement Management, Rafed UAE, Abu Dhabi, ARE; 11 Hematology, Tawam Hospital, Al Ain, ARE; 12 Clinical Pharmacy, Abu Dhabi Health Services Company (SEHA), Abu Dhabi, ARE; 13 Pediatrics/Genetics and Genomics, United Arab Emirates University, Al Ain, ARE; 14 Drugs and Medical Products Regulation, Department of Health, Abu Dhabi, ARE; 15 Health Service, Dubai Health Authority, Dubai, ARE; 16 Health Economics, Syreon Middle East, Cairo, EGY; 17 Health Economics, Syreon Research Institute, Budapest, HUN; 18 Health Technology Assessment, Semmelweis Univeristy, Budapest, HUN

**Keywords:** united arab emirates, reimbursement, rare disease, orphan drugs, multi criteria decision analysis, mcda

## Abstract

Background

The number of orphan drug approvals is currently increasing globally. This creates a significant burden on payers and healthcare systems. This study aimed to create a multi-criteria decision analysis (MCDA) tool for evaluating orphan drugs within the United Arab Emirates (UAE). The intended result of the tool is to provide evidence-based guidance to decision-makers in reimbursement and procurement decisions.

Methods

We conducted a literature search and local expert interviews to identify relevant preliminary criteria for the MCDA tool. Then we conducted a structured consensus-building session for healthcare experts and decision-makers in the UAE to develop the Emirati MCDA tool for orphan drugs. The experts voted for the criteria to be included in the tool and their ranking according to importance, as well as the weight of each criterion and its scoring function. To improve understanding and facilitate the voting process, experts were provided with a brief illustration of similar tools conducted in other countries before the voting sessions. Finally, the tool was developed in a Microsoft Excel sheet (Microsoft Corporation, Redmond, Washington, United States), and it was validated and tested based on real case studies, then it was fine-tuned accordingly based on the experts’ discussions. The final tool was provided to the attendees to guide their decisions in the reimbursement and procurement of orphan drugs.

Results

The created tool provides a score for each analyzed orphan drug based on its value. Ten criteria were included in the final MCDA tool. These were cost-effectiveness (25.1% of the weight), magnitude of health gain (20.1%), availability of therapeutic alternative (14.3%), disease severity (11%), budget impact (7.9%), disease rarity (5.6%), strength of clinical evidence (5.6%), burden on households (4.5%), indication uniqueness (3.2%), and patients’ age (2.6%).

Conclusions

Implementation of evidence-based healthcare necessitates assessing the fair value of each health technology. Addressing the high unmet medical needs and improving healthcare for patients with rare diseases are priorities within the UAE. The created Emirates MCDA tool for orphan drugs has the potential to help decision-makers implement value-based and evidence-based reimbursement decisions for orphan drugs.

## Introduction

Orphan drugs are used to treat or prevent rare diseases [1]. In the past, orphan drugs did not get sufficient attention from pharmaceutical companies [2,3] due to the small number of patients and several hurdles that innovators faced when developing an orphan drug, such as the heterogeneous patient populations, insufficient data, high uncertainty, and difficulty in recruiting patients for clinical trials [2,4]. Consequently, the return on investment for orphan drugs was less than for new technologies in common diseases.

To reduce the unmet need for such neglected diseases, some countries and regions with a special focus on pharmaceutical research and development (R&D) implemented regulatory and policy incentives for pharmaceutical companies to increase investment in the development of orphan drugs. These include the European Union, the United States, Japan, Australia, South Korea, Brazil, and India [5]. This resulted in a higher number of approved orphan drugs over the past few years [5,6], which in turn has led to increased pharmaceutical spending for treating orphan diseases despite their low prevalence [6].

United Arab Emirates (UAE) is committed to having early access to innovation and has ranked as a world leader in rapid drug approvals with its fast-track registration system, which has been implemented since 2018 [7]. This system aims to accelerate the registration process and eliminate long waiting times for innovative and orphan drugs [7]. In addition, the healthcare system in the UAE is rapidly moving towards health technology assessment (HTA) implementation [8,9]. This necessitates assessing the available technologies to make valid reimbursement decisions [10]. Although the UAE had the highest number of orphan drugs among countries in the Middle East North Africa (MENA) region, it also had the highest median annual prices for orphan drugs [11].

Additionally, the UAE has introduced mandatory health insurance where UAE nationals are covered by a government-mandated scheme "Thiqa". Through this scheme, all indicated therapies including highly priced newly innovative medicines and orphan drugs are fully covered [12]. UAE also has special treatment cost exemption programs for cancer patients receiving orphan drugs, including non-UAE nationals [13,14]. Covering high-cost orphan medications puts a financial burden on the healthcare scheme. This requires the presence of an accurate and comprehensive tool for assessment of the available technologies to ensure evidence-based decisions towards improved health outcomes.

Traditional value assessment frameworks may not capture all the benefits of orphan drugs, as they focus on health gain versus the incremental cost and neglect other benefits, such as response to an unmet medical need without alternative treatment options, or promoting equity [15,16]. Adding these criteria to evaluation frameworks should provide orphan drugs with a fair chance of proving their true societal value [16,17].

Multi-criteria decision analysis (MCDA) tools are tools that help in comparing different alternatives to provide a final score to each, based on several objective criteria. Scoring functions and weights are developed to set the importance of each decision criterion used [18]. Several value frameworks and MCDA tools have been recently developed to choose among alternatives or to help quantify benefits that are difficult to assess [19-22]. However, MCDA tools are not transferrable between different countries or settings; each should develop a tailored tool to cover its local needs [23]. Therefore, this study aimed to recommend an objective tool that helps decision-makers in providing an evidence-based value for orphan drugs in the UAE, and consequently make transparent and justifiable evidence-based decisions.

## Materials and methods

In light of the constrained expertise in the UAE for developing an MCDA tool, our methodology was informed by a guidance paper that was specifically tailored for developing MCDA tools in countries with limited human resources [24].

**Figure 1:**
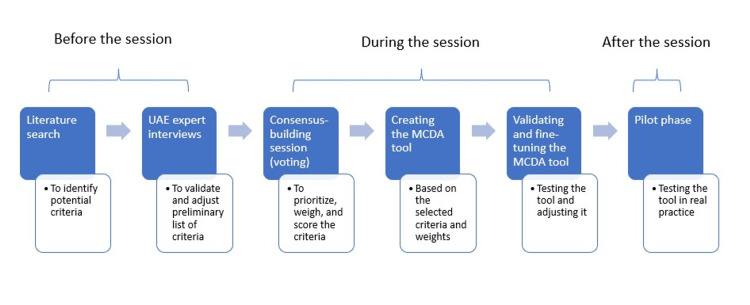
Steps to create the MCDA tool MCDA: multi-criteria decision analysis; UAE: United Arab Emirates

Creating the MCDA tool comprised several steps. Initially, a literature search was undertaken to identify potential criteria and scoring functions used for similar tools globally. This was followed by online interviews with UAE experts to refine and tailor the identified preliminary list of criteria to align with the UAE’s specific context. Next, we conducted a structured consensus-building session with stakeholders involved in UAE’s procurement and reimbursement to vote for prioritizing, weighting, and scoring the criteria. The stakeholders’ votes were used to inform the construction of the MCDA tool during this session. Stakeholders further validated the tool’s efficacy using test cases. After reviewing the outcomes of these test cases with the stakeholders, refinements were made to the tool based on their feedback. The tool was set for a pilot phase, with the possibility of additional adjustments before its formal deployment. The study steps are illustrated in Figure 1.

Preparation (before the session)

Following the guidance paper, we relied on previous MCDA tools and value frameworks to define the initial list of criteria. We complemented this with the experience of local experts who could advise on the country’s specific settings. For this, our research team first searched the literature to identify a pool of relevant criteria for inclusion in the MCDA tool. We searched PubMed and grey literature for published MCDA tools or reviews that report criteria used for the evaluation of orphan drugs globally, or relevant criteria for general MCDA tools. We used two domains for the search: MCDA and orphan drugs. The search term was based on these domains and their synonyms (“MCDA” OR “Value frameworks” OR “Multi-criteria decision analysis”) AND (“Orphan” OR “Rare disease”). The search was conducted in February 2022 and there was no limitation on the date of publication. We included any MCDA tool or value framework designed to evaluate the value of orphan drugs, detailing specific criteria names, with or without scoring functions.

For the grey literature sources, we were more focused on identifying criteria related to evaluating drugs in UAE, or countries with similar settings, so we included any identified MCDA tool or value framework that was conducted in UAE or its neighboring countries for drug evaluation. We used the identified studies and tools as guidance to help in creating the preliminary list of criteria, that are relevant and evaluable in the UAE.

The research team compiled all criteria used in these tools, removed duplicates, and merged similar criteria to have a preliminary list of criteria. Some of those criteria were nonrelevant to the UAE or were evaluated differently, so local reimbursement experts were interviewed to validate the eligibility of all the criteria and scoring functions in the local context. These criteria were adjusted and filtered during the interviews to provide a shortlist of the locally relevant criteria.

Each criterion was provided with a proposed scoring function. Scoring functions were initially identified from the literature search and were further adjusted or complemented through suggestions of the UAE experts. The experts advised on the specific settings in the UAE healthcare and reimbursement system.

This preliminary list aimed to give decision-makers in UAE a head start to creating their own MCDA tool. The proposed criteria and scoring functions were optional; decision-makers had the opportunity to add, remove, or edit any of the criteria or scoring details during the consensus-building session.

Structured consensus-building session

We conducted a structured consensus-building session among healthcare professional experts from the UAE to select and adjust the criteria for assessing orphan drugs. The session was conducted in Abu Dhabi, UAE. Eleven healthcare experts and decision-makers representing different public healthcare sector entities in the UAE participated in the structured consensus-building session on June 26, 2022. The inclusion criteria of the participating experts included having extensive experience (more than five years) in the tendering and reimbursement system in UAE, a good understanding of HTA concepts, and current employment in a public healthcare institution in UAE. Additionally, we also considered having representation of the various healthcare sectors within the UAE, to facilitate the utilization of the tool across healthcare organizations in the country. Due to the limited number of experts in these fields in UAE, we used convenience sampling to identify experts who fulfilled the inclusion criteria and were willing to participate in the session.

The participants came from several governmental and semi-governmental institutions in the UAE. Participants included three clinical experts from Tawam Hospital, two experts from Abu Dhabi Health Services Company (SEHA), two experts from the Abu Dhabi Department of Health (DoH), an expert from Dubai Health Authority (DHA), an expert from the UAE Ministry of Health and Prevention (MOHAP), an expert from United Arab Emirates University, and an expert from Rafed, the UAE’s leading healthcare supply chain.

The objectives of conducting the consensus-building session were to prioritize, and weigh the proposed criteria, and to validate the proposed scoring functions. During the session, participants ranked the criteria from the most important to the least important from the UAE perspective. The research team proposed preliminary scores for the included criteria. Participants validated the proposed scores and scoring functions by discussing if they adequately assessed the value of the compared drugs.

As a next step, criteria were weighted relative to each other using the Simple Multi-Attributable Rating Technique (SMART) & Swing method [25]. The session moderator first explained the SMART & Swing method in detail with a special focus on how weights were calculated and normalized. Then participants ranked the criteria and voted for the relative importance of each criterion compared to the next ranked criterion. The normalized results were shown to the participants, and then the use of the Microsoft Excel (Microsoft Corporation, Redmond, Washington, United States) tool was presented with a detailed explanation on how to calculate the final score of orphan drugs.

The created tool requires the user to choose the outcomes that match each drug’s specifications from the available options and to add the letter x at the corresponding cell. The tool then automatically calculates the final score of the drug. The tool allows four drugs to be tested simultaneously and calculates the score of each to be easily compared.

Technical steps

Voting was conducted anonymously on Mentimeter® online software (Mentimeter AB (publ), Stockholm, Sweden). Participants voted through their mobile phones during the consensus-building session. The MCDA tool was developed on Microsoft Excel, and the participants received the final tool after all results and voting were implemented. For each question, the average of the votes was calculated and used as the final result. Finally, the customized MCDA tool was built to assess orphan drugs in the UAE based on objective criteria that are locally relevant.

Validation and consistency of the developed tool

The Emirate MCDA tool was tested by the session participants through four test cases and concordance between the expected and observed results was assessed. The aim of the validation step was to test the tool’s functionality and to ensure it provided valid and realistic results for the cases that were prepared before the session. The concordance analysis was based on three predefined criteria: (i) Score (more than or less than 50 points out of 100); (ii) Rank order (ranks of the compared therapies); (iii) Score difference between highest and lowest comparator (at least 40 points).

The four test cases assessed for validation included a genetic curative therapy for an ultrarare disease, a highly effective chronic therapy for a rare disease, a high-quality symptomatic chronic therapy, and a lower-quality symptomatic chronic therapy. The expected results were that the genetic curative therapy and the highly effective chronic therapy should receive a score higher than 50, while the other two comparators should receive scores below 50 points. The expectation was also that the score ranks would be as follows: genetic curative therapy, highly effective chronic therapy, higher quality symptomatic chronic therapy, and finally the lower quality symptomatic chronic therapy. The final expected result was that the difference between the highest and lowest comparators should be at least 40 points.

## Results

Literature search results

Through the literature search, we identified four relevant previous summary papers on the evaluation criteria for innovative medicines and orphan drugs [17,19,22,26]. Additionally, we identified three unpublished MCDA tools that were developed in neighboring countries (Oman, Qatar, and Egypt). Although these three tools were not developed to evaluate orphan drugs, their criteria were still useful in preparing the preliminary list to ensure the tool's comprehensiveness. The criteria identified from the three MCDA tools and their details are presented in Appendix A.

Adjusting and filtering the criteria resulted in a preliminary list of 12 criteria. The preliminary list included indication uniqueness, availability of therapeutic alternative (unmet need), the average age of patients in clinical trials or real world, magnitude of health gain, disease rarity, disease severity, advancement of technology, manufacturing technology complexity, credibility and robustness of clinical evidence, budget impact, cost-effectiveness, and societal burden.

Criteria and scoring

The evaluation criteria proposed for the tool were categorized into disease-related, economic-related, and treatment-related criteria.

Disease-Related Criteria

Disease rarity: This criterion is employed to assess the uncommonness of a disease based on its prevalence. This criterion gives a higher score for diseases with exceptional rarity compared to more prevalent diseases. A "rare disease" was defined as one that affects less than five individuals per 10,000 in the population, while an "ultrarare disease" was defined as a disease affecting one or fewer individuals per 50,0000 in the population. This classification aligns with the approach used by the National Institute of Clinical Excellence (NICE) for their health technology appraisals [27]. Furthermore, an additional subgroup was incorporated: a rare subgroup of a common disease. This subgroup was defined as diseases affecting a group of patients who have a rare mutation or variant of a disease that is otherwise not rare.

Availability of a therapeutic alternative (unmet need): This criterion aims to assess if there are other treatments available for the disease. If there are no effective treatments, then the drug should receive a high score here, as it fulfills an unmet need, but if there are other similar drugs, then the assessed drug will not receive a high score as, although it still may help in treating the disease, it will be to a lower extent compared to another that has no alternatives.

Disease severity: This criterion aims to prioritize allocating higher scores to drugs that address more severe diseases. Severity is assessed through three subcategorizations to distinguish between different disease classes: (i) Acute or chronic nature of the disease; (ii) Life-threatening potential of the disease; (iii) Extent of disability or functional impairment caused by the disease. The definition of a chronic disease adheres to the criteria established by the Centers for Disease Control and Prevention (CDC) [28], while the level of disability (severe invalidity) was defined as the substantial impairment of capacities central to individuals’ functional ability in the society [29].

Burden on households: This criterion aims to provide a higher score for diseases that have a high financial burden (other than direct medical costs) on patients and caregivers compared to other diseases with a limited burden (i.e., diseases that require expensive home modifications, high out-of-pocket payments, or diseases requiring the presence of permanent formal or informal caregivers will receive a higher score in this criterion compared to diseases without this type of burden).

Economics-Related Criteria

Budget impact: This criterion takes into consideration both the number of patients and the cost of treatment. If the expected budget impact of an orphan drug increases as a proportion of the annual drug budget, the score becomes lower. Although rare diseases have a low prevalence, budget impact is still a relevant criterion for decision-makers, because most orphan drugs are very costly, and their number is growing rapidly. Therefore, the total budget for orphan drugs may eventually consume a substantial part of the overall pharmaceutical budget, which is a global concern for health systems.

Cost-effectiveness: This criterion aims to facilitate value-based pricing, so it provides a higher score for more cost-effective drugs. Assessment of cost-effectiveness is commonly tied to the gross domestic product (GDP) per capita in a specific country [30]. Although most countries are willing to make some exceptions regarding the cost-effectiveness thresholds for orphan drugs [16,31,32], it is still important for decision-makers to evaluate the cost-effectiveness of the drug and use it as one of the criteria in the MCDA. Otherwise, a drug that meets the other evaluation criteria in the MCDA could be approved at any price, regardless of the value it provides.

Treatment-Related Criteria

Magnitude of health gain: This criterion involves providing a higher score for treatments that provide more health benefits. For example, curative therapies should receive the highest score compared to symptom relief therapies or temporary therapies.

Credibility and robustness of clinical evidence: This criterion provides a higher score for treatments with robust evidence compared to other treatments which may have no or weak evidence of their efficiency. To evaluate this criterion, it is imperative to review the study designs for providing clinical data for each compared drug. Here, data derived from randomized controlled trials are prioritized over data obtained from non-randomized trials or single-arm studies. Also, data from long-term real-world studies are prioritized over data from shorter real-world studies [33,34].

Indication uniqueness: This criterion acknowledges a higher score for limited return on investment for manufacturers of medicines with a unique orphan indication. Other orphan drugs that treat more than one orphan indication or even have indications in common diseases receive less score in this criterion.

Advancement of technology: This criterion provides a higher score for first-in-class medicines compared to medicines with regularly used mechanisms of action.

Manufacturing complexity: This criterion provides a higher score for drugs that have complex manufacturing processes, like biotechnology and gene therapy drugs.

Average age of patients in clinical trials or the real world: This criterion provides a higher score for treatments assigned to younger patients to acknowledge the importance of future generations in our societies. Younger patients usually exhibit a higher disease burden, as quantified by disability-adjusted life years (DALYs), due to a more significant loss of life years from diseases compared to older patients [35]. Consequently, this enhances the value proposition of health technologies targeted to this population.

Structured consensus-building session outcomes

Ranking

Voting was conducted to prioritize the criteria from most important to least important according to their importance from the UAE perspective. “Cost-effectiveness”, “Magnitude of health gain”, and “Unmet medical need” were selected as the three most important criteria. “Disease rarity” and “Credibility and robustness of clinical evidence” were considered equally important criteria. The final ranking of criteria is shown in Table 1.

**Table 1:** Orphan MCDA criteria ranked according to importance ^*^Disease rarity and credibility and robustness of clinical evidence criteria had the same weight, so they share the 6th rank among the criteria. QALY: quality-adjusted life year; MCDA: multi-criteria decision analysis

Rank	Criterion
1	Cost-effectiveness (Cost/QALY)
2	Magnitude of health gain
3	Therapeutic alternative (unmet need)
4	Disease severity (expected QALYs without therapy)
5	Budget impact
6	Disease rarity
6*	Credibility and robustness of clinical evidence
7	Burden on households (patients & caregivers)
8	Indication uniqueness
9	Average age of patients in clinical trials or real-world

Selecting the Number of Criteria

During the consensus-building session, participants were asked to vote for the final number of criteria to be included in the tool. Six out of 11 votes voted to keep 10 criteria, and consequently, only 10 criteria were left, while the others were excluded based on the ranking voting. Two criteria were excluded as they received the lowest ranks: “Advancement of technology” and “Manufacturing complexity”.

Scoring Functions

Scoring functions explain how each medication could score in a specific criterion (e.g., A drug fulfilling the requirement of a specific criterion achieves a higher score in that criterion compared to a drug that fulfills half the requirements or does not fulfill it at all). Table 2 shows the scoring function and scores for each criterion.

**Table 2:** Scoring functions ^*^A significant household burden on patients or caregivers was defined as evidence for a financial burden due to lost productivity or time as an effect of the disease, reported in at least one peer-reviewed study. QALY: quality-adjusted life year; RCT: randomized controlled trial; GDP: gross domestic product; MCDA: multi-criteria decision analysis

Criterion	Scoring function (Outcome)	Score
Indication uniqueness	One unique orphan indication	100%
	Two orphan indications	50%
	More than two orphan indications	25%
	Non-orphan indications	0%
Therapeutic alternative (unmet need)	No effective treatments are available	100%
	Less effective treatments are available	50%
	Similarly effective treatments are available	0%
Magnitude of health gain	Curative therapy (no need for further therapy)	100%
	Long-term remission on therapy (therapy until progression)	75%
	Duration of benefits is 1-5 years	50%
	Symptom relief or short-term benefit	0%
Average age of patients in clinical trials or real world	Pediatrics (0-16 years)	100%
	Young adults (17-30 years)	60%
	Middle aged adults (31-65 years)	30%
	Old age adults (above 65 years)	0%
Disease rarity	Ultrarare disease	100%
	Rare disease	50%
	Rare subgroup of a common disease	0%
Disease severity (expected QALYs without therapy)	Chronic life threatening	100%
	Acute life threatening	80%
	Chronic with severe invalidity	60%
	Acute with severe invalidity	40%
	Other chronic diseases	20%
	Other acute diseases	0%
Credibility and robustness of clinical evidence	Supportive RCT and real-world evidence	100%
	Supportive RCT with at least 1 year follow-up	75%
	Supportive RCT with <1 year follow-up	50%
	Single arm phase 2 study	0%
Budget impact	Below 0.01% of annual drug budget	100%
	Between 0.01-0.05% of annual drug budget	75%
	Between 0.05-0.10% of annual drug budget	50%
	Between 0.1-0.3% of annual drug budget	25%
	Above 0.3% of annual drug budget	0%
Burden on households (patients and caregivers)	Burden on patients or caregivers > direct medical cost	100%
	Significant burden on patients or caregivers*	75%
	No evidence on household burden	0%
Cost-effectiveness (cost/QALY)	Below 1x GDP per capita	100%
	Between 1-2x GDP per capita	80%
	Between 2-3x GDP per capita	60%
	Between 3-5x GDP per capita	40%
	Between 5-10x GDP per capita	20%
	Above 10x GDP per capita	0%

Weighting

Participants voted for the relative weight of each criterion compared to the next ranked criterion. The votes were then normalized to 100% (see Table 3).

**Table 3:** Summary of the ranking and weights of criteria in the Emirate MCDA tool QALY: quality-adjusted life year; MCDA: multi-criteria decision analysis

Criteria	Ranking	Weight increase	Normalized weight
Cost-effectiveness (Cost/QALY)	1	25.0%	25.1%
Magnitude of health gain	2	40.0%	20.1%
Therapeutic alternative (unmet need)	3	30.0%	14.3%
Disease severity (expected QALYs without therapy)	4	40.0%	11.0%
Budget impact	5	40.0%	7.9%
Disease rarity	6	0.0%	5.6%
Credibility and robustness of clinical evidence	6	25.0%	5.6%
Burden on households (patients & caregivers)	7	40.0%	4.5%
Indication uniqueness	8	25.0%	3.2%
Average age of patients in clinical trials or real-world	9	25.0%	2.6%

Validation and concordance of the MCDA tool

The results of the case studies testing proved that the tool works efficiently and that it provides a fairly low score for orphan drugs that provide minimal benefits, low quality, or both, while it positively discriminates therapies that have a large health benefit. The framework consistently provided results in line with national experts’ expectations.

The genetic curative therapy ranked first with 69.2 points, the highly effective chronic therapy ranked second with 59.9 points, the high-quality symptomatic treatment ranked third with 42.0 points, and the lower-quality symptomatic chronic treatment ranked last with 29.5 points. These results showed 100% concordance with the experts’ expectations in the score and rank validation criteria. However, the concordance criterion was not met for the score difference criterion, as the difference between the highest and lowest comparators was 39.7, which is less than the predefined threshold of 40. However, there was a consensus from the experts to use this tool despite this minor discordance, as they believed the tool provided logical results.

Final tool

At the end of the consensus-building session, the research team compiled the voting results into the Microsoft Excel MCDA tool and adjusted the tool in a way that could be easily used by the users. To use the final Microsoft Excel tool, the user should choose the outcomes that match each drug’s specifications from the available options. The tool then automatically calculates the final score of the drug, and it allows for the comparison of multiple drugs. The final tool is provided in Appendix B. There was consensus among the session participants that a pilot phase of using the tool in the healthcare system should be implemented, and that the tool can be readjusted after that if required.

## Discussion

All healthcare systems aim to allocate the available resources to the interventions that provide the most benefits to the patients. The healthcare experts in UAE thought of using an MCDA tool to help them assess the value of orphan drugs, as these are currently increasing in the market, and they usually come with high prices. Although orphan drugs may not be cost-effective when assessed with traditional value assessment tools, experts in the UAE believe that the full societal value of orphan medicines should be evaluated based on an extended value framework, which necessitates the development of an MCDA tool for repeated use.

The MCDA tool created can be used by decision-makers in UAE to decide upon the orphan drugs that deserve to be reimbursed based on the final score each drug will accumulate.

In this tool, “cost of the drug” was not added as a separate criterion, as it was considered implicitly within the cost-effectiveness as well as the budget impact criterion. This is different from other traditional value frameworks and MCDA tools in terms of the fact that it doesn’t intend to provide a single winner or compare different alternatives. Instead, the created tool aims to provide a score on a scale of 100, which would act as a supportive tool for decision-makers in reimbursement decisions of orphan drugs, where the higher the score, the higher the probability this drug should be reimbursed, and vice versa. In the future after gaining experience with the developed tool in several real-world cases followed by finetuning of the tool a cutoff threshold could potentially be developed as an explicit guidance for decision making.

The Emirate MCDA tool developed by healthcare professional experts in UAE takes into consideration traditional value assessment criteria such as cost-effectiveness, budget impact, and health gain. It also adds criteria that are specific for orphan drugs such as indication uniqueness, rarity of the disease, and availability of therapeutic alternatives. Additionally, criteria that can be applied to any health technology in common diseases were included, such as burden on households, credibility of evidence, disease severity, age of patients, and magnitude of health gain. This creates a comparison of the full societal value of orphan drugs in rare diseases, which would have a limited chance for reimbursement based on traditional value assessment.

Altogether, three orphan-related criteria constituted 23.1% of the total weight. This result provided an additional benefit (score) for a drug that treats a less prevalent disease, but at the same time, it would still keep emphasis on other general factors that are indispensable in decision-making.

Our results are concurrent with orphan MCDA tools published previously. The most common criteria in similar tools like “need for intervention”, “disease severity” and “cost-effectiveness” [22] are also present in the tool we created. However, some studies had a comparative efficacy/effectiveness criterion, other than the cost-effectiveness criterion [19,22]. This may create an overlap and double counting of the effectiveness effect. Our experts were aware of that point, and they insisted that the “cost-effectiveness” criterion accounts for both effects of cost and effectiveness. So, they agreed not to add other cost-related or effectiveness-related criteria to prevent overlap.

The two criteria that were excluded (“advancement of technology” and “manufacturing complexity“) aimed to reward manufacturers for their investment. However, the experts agreed that by using the created tool, manufacturers would be rewarded if they provided a drug that helps to improve health outcomes in general, disregarding its manufacturing costs. The excluded criteria were also not included in most of the MCDA tools assessed by Baran-Kooiker et al. in their systematic review of MCDA tools for orphan drugs [22].

The analysis showed that the Emirate MCDA tool is largely consistent with national experts' expectations in evaluating the value of therapies for rare diseases. While the tool effectively distinguished between therapies of varying benefits and appropriately ranked them, it did not fully meet the criteria for score differences between the highest and lowest-benefit therapies. This suggests the need for further calibration of the tool or reconsideration of the score difference criterion to enhance its applicability and accuracy in real-world decision-making.

The experts agreed that the developed Emirates MCDA tool may still be fine-tuned after trying it in the healthcare system. In other words, the initial tool should be tested in a pilot period after which it has to be revised according to the initial experience. Subsequently, room for improvement should be considered on a periodical basis (e.g., every two or three years).

Limitations

The relatively low number of participants in the structured consensus-building session was a limitation of this study. This was mainly due to the scarcity of local experts in HTA, especially since the UAE was in an early stage of HTA implementation at the time of the study.

Another limitation was that some of the proposed criteria were not mutually exclusive. This means that one factor could be accounted for through more than one criterion, and its effect is double counted in the tool. For this, as much as possible, experts excluded criteria that were duplicated, and those that measure the same factor for an orphan drug; however, some other factors are difficult to isolate and will therefore be accounted for in more than one criterion, like cost, for instance, is accounted for in the cost-effectiveness and the budget impact criteria. However, experts were aware of that point and they knew that each criterion aimed to assess an important aspect of the orphan drug, and they were aware of the double counting, so they considered this while voting for the weights of those criteria.

A limitation of this study lies in the methodology for conducting the MCDA, which is mainly related to the consensus-building session results and experts’ opinions. This methodology might not be rigorous and there is a possible inefficiency in the results; however, the pilot tool aims to mitigate this limitation and to test how the tool performs before its formal implementation. If any gaps or inefficiencies are spotted, this tool could be adjusted to ensure the results are fair and robust. Furthermore, the tool currently only provides a score that can give insight to decision-makers but still doesn’t have a cutoff threshold against which scores can be benchmarked. However, this can be developed in the future.

The concordance analysis has a few limitations as well; the reliance on just four case studies may not comprehensively represent the tool's applicability across diverse healthcare scenarios, reducing the generalizability of the results. Also, the use of expert-defined expectations introduces subjectivity, as different experts may have varying opinions on what constitutes significant benefits, affecting concordance outcomes. However, even with these limitations, the concordance analysis still provided confidence that the tool provides logic and consistent results.

## Conclusions

Policymakers apply special incentives to positively discriminate orphan medicines for patients who suffer from rare diseases. In some countries, orphan drugs are excluded from the mandatory cost-effectiveness criteria, which means that the economic value of orphan drugs is not taken into account in policy decisions. Implementation of evidence-based healthcare necessitates the value judgment of each technology with high priority. Since improving the management of patients with rare diseases is among the healthcare priorities in UAE, we developed a recommendation for an MCDA tool to help decision-makers assess the full societal value of orphan drugs. This tool has the potential to help decision-makers make evidence-based reimbursement decisions for new technologies in rare diseases.

## Appendices

Appendix A

**Table 4:** List of criteria identified through grey literature *Ibrahim Alrashdi, Ahmad Fasseeh, Zoltán Kaló, et al. Multi-Criteria Decision Analysis for Out-of-Patent Pharmaceuticals Purchasing in Oman; April 2019 **Hisham Nasr, Ahmed Abdelgawad, Ahmad Fasseeh, et al. Multi-Criteria Decision Analysis (MCDA) Out-of-Patent Pharmaceuticals Workshop for Qatar; April 2019 ***Amany Farag, Ahmad Fasseeh, Baher Elezbawy, et al.  Egypt University MCDA Tool Unification; February 2019 MCDA: multicriteria decision analysis

	Oman MCDA tool*	Qatar MCDA tool**	Egypt MCDA tool***
1	Equivalence with the reference (original) product	Equivalence with the reference (original) product	Use in reference countries
2	Method of administration	Product manufacturing quality certification	Bioequivalence
3	API manufacturing quality certification	API manufacturing quality certification	Quality assurance
4	Product manufacturing quality certification	Pharmaceutical product stability	Years of experience in local market
5	Supply reliability	Macroeconomic benefit/ Manufacturer local investment	Previous use in tenders
6	Refund/Replace for expired products	Refund/Replace for expired products	Previous use in hospitals
7	Pharmaceutical product stability	Supply reliability	Previous experience with the manufacturer
8	Pharmacovigilance	Pharmacovigilance	Supply reliability

Appendix B

**Table 5:** Emirati MCDA tool for orphan drugs in the United Arab Emirates MCDA: multicriteria decision analysis

Criterion	Weight	Scoring options	Score	Comparator 1	Comparator 2	Comparator 3	Comparator 4
Cost-effectiveness (cost/QALY)	25.1%	Below 1x GDP per capita	100%				
		Between 1-2x GDP per capita	80%				
		Between 2-3x GDP per capita	60%				
		Between 3-5x GDP per capita	40%				
		Between 5-10x GDP per capita	20%				
		Above 10x GDP per capita	0%				
Magnitude of health gain	20.1%	Curative therapy (no need for further therapy)	100%				
		Long-term remission on therapy (therapy until progression)	75%				
		Duration of benefits is 1-5 years	50%				
		Symptom relief or short-term benefit	0%				
Therapeutic alternative (unmet need)	14.3%	No effective treatments are available	100%				
		Less effective treatments are available	50%				
		Similarly effective treatments are available	0%				
Disease severity (expected QALYs without therapy)	11.0%	Chronic life threatening	100%				
		Acute life threatening	80%				
		Chronic with severe invalidity	60%				
		Acute with severe invalidity	40%				
		Other chronic diseases	20%				
		Other acute diseases	0%				
Budget impact	7.9%	Below 0.01% of annual drug budget	100%				
		Between 0.01-0.05% of annual drug budget	75%				
		Between 0.05-0.10% of annual drug budget	50%				
		Between 0.1-0.3% of annual drug budget	25%				
		Above 0.3% of annual drug budget	0%				
Disease rarity	5.6%	Ultrarare disease	100%				
		Rare disease	50%				
		Rare subgroup of a common disease	0%				
Credibility and robustness of clinical evidence	5.6%	Supportive RCT and real-world evidence	100%				
		Supportive RCT with at least 1 year follow-up	75%				
		Supportive RCT with <1 year follow-up	50%				
		Single arm phase 2 study	0%				
Burden on households (patients & caregivers)	4.5%	Burden on patients or caregivers > direct medical cost	100%				
		Significant burden on patients or caregivers	75%				
		No evidence on household burden	0%				
Indication uniqueness	3.2%	One unique orphan indication	100%				
		Two orphan indications	50%				
		More than two orphan indications	25%				
		Non-orphan indications	0%				
Average age of patients in clinical trials or real world	2.6%	Paediatrics (0-16 years)	100%				
		Young adults (17-30 years)	60%				
		Middle aged adults (31-65 years)	30%				
		Old age adults (above 65 years)	0%				
Total score				0.0	0.0	0.0	0.0

## References

[1] (2022). EC/847/2000 Commission Regulation (EC) No 847/2000 of 27 April 2000 laying down the provisions for implementation of the criteria for designation of a medicinal product as an orphan medicinal product and definitions of the concepts ‘similar medicinal prod. https://www.gmp-compliance.org/guidelines/gmp-guideline/ec-847-2000-commission-regulation-ec-no-847-2000-of-27-april-2000-laying-down-the-provisions-for-implementation-of-the-criteria-#:~:text=established%20a%20hyperlink.-,EC%2F847%2F2000%20Commission%20Regulation%20(EC)%20No%20847,the%20concepts%20'similar%20medicinal%20prod.

[2] Fonseca DA, Amaral I, Pinto AC, Cotrim MD (2019). Orphan drugs: major development challenges at the clinical stage. Drug Discov Today.

[3] Aronson J (2006). Rare diseases, orphan drugs, and orphan diseases. BMJ.

[4] Nestler-Parr S, Korchagina D, Toumi M (2018). Challenges in research and health technology assessment of rare disease technologies: report of the ISPOR Rare Disease Special Interest Group. Value Health.

[5] (2022). Rare Diseases: Regulatory incentives for development of orphan drugs - the United States & Europe. https://credevo.com/articles/2019/12/15/rare-diseases-regulatory-incentives-for-development-of-orphan-drugs-us-europe/.

[6] Zimmermann BM, Eichinger J, Baumgartner MR (2021). A systematic review of moral reasons on orphan drug reimbursement. Orphanet J Rare Dis.

[7] (2022). Minister of Health and Prevention issues ministerial decree for the registration of innovative medicines and rare drugs. https://mohap.gov.ae/en/media-center/news/25/1/2018/minister-of-health-and-prevention-issues-ministerial-decree-for-the-registration-of-innovative..

[8] Al Suwaidi A (2016). The future Of HTA in evolving health care systems: developing HTA strategy for the United Arab Emirates. Val Health.

[9] Fasseeh A, Karam R, Jameleddine M (2020). Implementation of health technology assessment in the Middle East and North Africa: comparison between the current and preferred status. Front Pharmacol.

[10] (2022). Health Technology Assessment Process: Fundamentals. https://toolbox.eupati.eu/resources/health-technology-assessment-process-fundamentals/.

[11] Achour L, Hanna E, Chachoua L, Dabbous M, Toumi M (2018). PSY59-orphan drugs prices comparison in Middle East North Africa (MENA) region. Val Health.

[12] (2022). Thiqa programme benefits and eligibility. https://www.thiqa.ae/en/about-thiqa-programme/thiqa-programme-network.

[13] Abu-Gheida IH, Nijhawan N, Al-Awadhi A, Al-Shamsi HO (2022). General oncology care in the UAE. Cancer in the Arab World.

[14] Al-Shamsi HO (2022). The state of cancer care in the United Arab Emirates in 2022. Clin Pract.

[15] McQueen RB, Inotai A, Zemplenyi A, Mendola N, Németh B, Kalo Z (2024). Multistakeholder perceptions of additional value elements for United States value assessment of health interventions. Value Health.

[16] Blonda A, Denier Y, Huys I, Simoens S (2021). How to value orphan drugs? A review of European value assessment frameworks. Front Pharmacol.

[17] Jakab I, Németh B, Elezbawy B (2020). Potential criteria for frameworks to support the evaluation of innovative medicines in upper middle-income countries—a systematic literature review on value frameworks and multi-criteria decision analyses. Front Pharmacol.

[18] P Hansen, N Devlin (2019). Multi-criteria decision analysis (MCDA) in healthcare decision-making. Oxford Research Encyclopedia of Economics and Finance.

[19] Zelei T, Mendola ND, Elezbawy B, Németh B, Campbell JD (2021). Criteria and scoring functions used in multi-criteria decision analysis and value frameworks for the assessment of rare disease therapies: a systematic literature review. Pharmacoecon Open.

[20] Farghaly MN, Al Dallal SA, Fasseeh AN (2021). Recommendation for a pilot MCDA tool to support the value-based purchasing of generic medicines in the UAE. Front Pharmacol.

[21] Abdullah AH, Holtorf AP, Al-Hussaini M, Lemay J, Alowayesh M, Kaló Z (2019). Stakeholder driven development of a multi-criteria decision analysis tool for purchasing off-patent pharmaceuticals in Kuwait. J Pharm Policy Pract.

[22] Baran-Kooiker A, Czech M, Kooiker C (2018). Multi-criteria decision analysis (MCDA) models in health technology assessment of orphan drugs—a systematic literature review. Next steps in methodology development?. Front Public Health.

[23] Kim DD, Bacon RL, Neumann PJ, Culyer A (2019). Assessing the transferability of economic evaluations: a decision framework. Non-communicable Disease Prevention: Best Buys, Wasted Buys and Contestable Buys.

[24] Inotai A, Nguyen HT, Hidayat B (2018). Guidance toward the implementation of multicriteria decision analysis framework in developing countries. Expert Rev Pharmacoecon Outcomes Res.

[25] Németh B, Molnár A, Bozóki S, Wijaya K, Inotai A, Campbell JD, Kaló Z (2019). Comparison of weighting methods used in multicriteria decision analysis frameworks in healthcare with focus on low- and middle-income countries. J Comp Eff Res.

[26] Guarga L, Badia X, Obach M (2019). Implementing reflective multicriteria decision analysis (MCDA) to assess orphan drugs value in the Catalan Health Service (CatSalut). Orphanet J Rare Dis.

[27] Clarke S, Ellis M, Brownrigg J (2021). The impact of rarity in NICE's health technology appraisals. Orphanet J Rare Dis.

[28] (2022). National Center for Chronic Disease Prevention and Health Promotion (NCCDPHP): About chronic diseases. https://www.cdc.gov/chronicdisease/about/.

[29] Kolasa K, Zwolinski KM, Kalo Z, Hermanowski T (2016). Potential impact of the implementation of multiple-criteria decision analysis (MCDA) on the Polish pricing and reimbursement process of orphan drugs. Orphanet J Rare Dis.

[30] Chi YL, Blecher M, Chalkidou K (2020). What next after GDP-based cost-effectiveness thresholds?. Gates Open Res.

[31] Fasseeh A, Elezbawy B, Korra N (2022). HPR180 eligibility of orphan drugs for preferential reimbursement in Egypt. Value in Health.

[32] (2024). Modifications to the ICER value assessment framework for treatments for ultra-rare diseases. Modifications to the Icer Value Assessment Framework for Treatments for Ultra-rare Diseases.

[33] Kim HS, Lee S, Kim JH (2018). Real-world evidence versus randomized controlled trial: clinical research based on electronic medical records. J Korean Med Sci.

[34] Chodankar D (2021). Introduction to real-world evidence studies. Perspect Clin Res.

[35] Salomon JA (2014). Disability-adjusted life years. Encyclopedia of Health Economics.

